# Adding meaningful distal action effects in feature binding

**DOI:** 10.3758/s13414-025-03092-9

**Published:** 2025-05-20

**Authors:** Nicolas D. Münster, Philip Schmalbrock, Christian Beste, Alexander Münchau, Christian Frings

**Affiliations:** 1 Department of Psychology, Cognitive Psychology, University of Trier, Universitätsring 15, D54296 Trier, Germany; 2 Institute for Cognitive & Affective Neuroscience (ICAN), University of Trier, Trier, Germany; 3 Department of Child and Adolescent Psychiatry, Cognitive Neurophysiology, Faculty of Medicine, TU Dresden, Dresden, Germany; 4 Institute of Systems Motor Science, University of Lübeck, Lübeck, Germany

**Keywords:** Feature binding, Action effects, Event file termination

## Abstract

Event files that bind features of stimuli, responses, and action effects figure prominently in contemporary views of action control. When a feature repeats, the previous event file including this feature is retrieved and can then influence current performance. It is still unclear, however, what terminates an event file. A tacit assumption is that registering the distal (e.g., visual, or auditory) sensory consequences of an action (i.e., the “action effect”) terminates the event file, thereby making it available for retrieval. Yet recently Frings et al. (2023) tested different distal action-effect conditions in standard stimulus–response (S-R) binding tasks and observed no modulation of S-R binding effects. It is conceivable though that the impact of distal action effects on feature binding hinges on the action mode of agents with intention-based mode (as compared to a stimulus-based mode) of action particularly emphasizing the possible impact of a meaningful distal action effect. Thus, we analyzed semantically meaningful distal action effects (participants switched simulated light bulbs on and off) in three experiments (*N*_total_ = 181). We found no clear impact of meaningful additional action effect presence or contingency on feature binding effects. The present study thus corroborates the notion that especially S-R binding tasks induce a stimulus-based action mode, in which proximal action effects are used for event file termination.

**Supplementary Information:**

The online version contains supplementary material available at 10.3758/s13414-025-03092-9.

## Introduction

When you enter a dark room, you typically press the light switch on the wall to switch on the light. Usually, the light is then switched on and your intention to illuminate the room has been successfully realized. When you leave the room, you press the light switch again and the light goes off.

An action episode like this usually comprises the execution of an action depending on certain stimulus and context features and is driven by an intended action goal (here: a lit room). Consequently, action control involves the feature codes (i.e., representations) of stimulus and response features that are included in the intended action (Elsner & Hommel, 2004; Frings et al., 2024). These feature codes are assumed to be functionally equivalent, meaning that action and perception of a certain event share a *common code* (Prinz, 1990; Hommel, 2001). This is reflected in ideomotor learning, according to which the representation of an intended action effect is used to select a certain action (Herwig & Waszak, 2012).

Following the theory of event coding (TEC, Hommel et al., 2001), all the features present in an action episode (of stimuli, responses, and action effects) are temporarily bound and stored in a so-called event file (Frings et al., 2024; Hommel, 1998, 2004). Due to bidirectional associations between these features, it is further assumed that the presentation of one or more of the features contained in the event file is sufficient to retrieve the whole event file in a following episode. This becomes apparent when the following episode does not completely match the first episode: The retrieved event file then interferes with the newly built event file. This causes so-called partial repetition costs in the form of longer reaction times or higher error rates compared to trials with full feature change/repetition (note that in the case of a complete mismatch, there is no interference, since no retrieval takes place). This has been corroborated using neurophysiological data (e.g., Beste et al., 2023; Eggert et al., 2022; Rawish et al., 2024). In contrast, a benefit (or at least no costs) is assumed for the exact repetition of an action episode’s feature configuration.

Experimentally, a prime-probe design is typically used to construct various trial types to realize these different event file relations: response repetitions and response changes are combined with repetitions and changes of specific stimulus features. These full/partial changes/repetitions of stimuli and responses result in performance costs and benefits, the relation of which is often integrated into a common feature binding effect score,^1^ such as the stimulus–response (S-R) binding effect (Henson et al., 2014) or the distractor-response binding effect (DRB, Frings et al., 2007).

In these paradigms, the dynamic management of stimulus-based event files is analyzed as it is assumed that binding and retrieval processes both contribute independently to the observed binding effects (see the binding and retrieval in action control [BRAC] framework, Frings et al., 2020). Such feature-binding tasks usually involve manipulating the presentation of the stimuli to which a response is required, for example, through the stimuli’s temporal occurrence (Frings & Moeller, 2012), their salience (Schmalbrock et al., 2021), their similarity to other stimuli (Singh et al., 2016), or their perception as figure or ground (Schmalbrock & Frings, 2022). However, the role of action *effects* is rarely examined in these tasks – here, the focus is on the stimuli to which a response is made and the response.

Yet recently, Frings et al. (2023) raised the question of how action effects contribute to the formation of event files in standard S-R binding tasks. Here it was assumed that an action effect might be necessary to terminate the event file as the action might only then be perceived as finished. Before the perceived termination of the event file, it should continue to be written into the old event file – retrieval of this event file by the features of a new episode would therefore not yet be possible.

This view is supported by recent findings regarding the segmentation of experiences: It was found that, in episodic memory, the continuous stream of perception is divided into mnemonic episodes by cognitive boundaries (Zheng et al., 2022). These boundaries could be detected at a neuronal level, determining what is retrieved together as an *episode*. Event file termination could therefore be a mechanism that reflects this formation of cognitive boundaries – and, possibly, action effects play a critical role in this process.

However, on the one hand, all paradigms involving keypresses include effects, such as the disappearance of a stimulus following a keypress. Hence, there is the tacit assumption (inherent in many previous papers) that action effects like the disappearance of a stimulus after pressing a key terminate the event file, rendering it accessible for retrieval in the next display. On the other hand, manipulations trying to avoid this by *not* making the stimuli disappear immediately by pressing a button (e.g., Hommel, 1998) still cause S-R binding effects – that is, they suggest S-R binding effects are not entirely reliant on such *external* action effects.

To approach the question of whether and how action effects are necessary for the termination of an event file, it is important to stress that there are different types of action effects. Action effects can be separated into *proximal* and *distal,* that is, body-related effects and environment-related effects (Pfister, 2019) with the former being perceived directly by the body, for example, the proprioceptive or the tactile feedback (Wolpert et al., 1995). Environmental-related effects are naturally those that cause a perceivable change in the environment. Therefore, most keypress-based paradigms include both body-related/proximal and environment-related/distal effects, that is, the haptic perception of the finger pressing the button as well as the disappearance of the stimulus.

In the above-mentioned study by Frings et al. (2023), a simple, arbitrary visual or auditory distal action effect was added (to a variant of the S1R1-S2R2 task,^2^ Hommel, 1998) and tested for its influence on S-R binding effects. Critically, no influence on S-R binding effects was found by adding any distal action effect to the S-R binding task. The authors concluded that only proximal, but not distal action effects played a role in the termination of an event file.

This reveals another factor that might influence event file termination: Is an action represented via its action goal or as a mere reaction to a trigger stimulus? This differentiation was proposed by Kunde et al. (2007). Here, one can separate action anticipations that are aimed at and planned as the anticipated perception of a change in the environment (i.e., the effect) from actions that are executed as soon as the environmental conditions are necessary to produce a certain effect. In the latter case, the occurrence of this certain condition in the environment (experimentally, e.g., a target stimulus) is sufficient for the execution of the action.

How an action is represented depends on the state or *mode* in which the action is performed. Herwig and Waszak (2012) suggest an *intention-based* and a *stimulus-based* action mode. In the latter, the action goal corresponds to the response to a stimulus, and not to the production of a distal action effect. Assuming that event file termination follows the reaching of the intended action goal, the distal action effect should be irrelevant when a response is made in the stimulus-based action mode. Critically, in most of the established cognitive psychology paradigms based on arbitrary stimulus–response-effect combinations, this stimulus-based action mode is induced through the task: A stimulus (the target) appears and thus indicates the environmental condition that determines the selection and execution of the action. Here, the response is the relevant marker that terminates the event file. Consequently, if the intention-based action mode is activated, the distal action effect should become relevant for marking the achievement of the action goal, and, therefore, event file termination.

A way to modulate the action mode is to use semantically meaningful effects and instruct the participants to create those effects with their actions. Hommel (1993), for example, was able to show a shift from a stimulus-based to an intention-based mode for the Simon effect: The effect reversed depending on whether the instruction emphasized the reaction to a stimulus (press the left or right button) or the intended production of an environmental/distal action goal (switching on the left or right lightbulb). This corresponds to Herwig and Waszak (2012), stating that action effect associations are more likely to affect behavior in actions that are performed in an intention-based mode of action.

### The present study

As in Frings et al. (2023), we were interested in whether feature binding effects would be affected by the presence or the contingency of an additional distal action effect. But in contrast to Frings et al. (2023), where arbitrary effects were used without any particular semantic connotation, we used a salient and semantically meaningful action effect (simulated lightbulbs) that is overlearned from everyday life. By doing so, we aimed to induce an intention-based action mode (cf., Herwig & Waszak, 2012), in which an action would be carried out with the focus on environmental changes (cf., Kunde et al., 2007) instead of a reaction triggered by the perception of a certain execution condition (the target stimulus). By manipulating distal action effects in various ways, we aimed to modulate event file termination: Insufficient termination should hamper event file retrieval, causing weaker feature binding effects.

For this purpose, we used simulated light bulbs as additional distal action effects and instructed participants that the response keys would work as light switches. To make the experience of the effect more realistic, we adapted response keys such that they worked like real switches, meaning that the light bulb was switched on when the corresponding button was first pressed and remained switched on until the button was pressed again ("long-lasting"action effect).

In a series of three experiments, we manipulated additional distal action effect presence (Experiment 1), contingency (Experiment 2), and type (Experiment 3). By contrasting the long-lasting action effect with different comparison conditions, we attempted to modulate the influence of distal effects on the strength of feature binding effects mediated by event file termination. In Experiment 1, we tested the long-lasting distal action effect in contrast to an effectless condition. Here, we (trial-wise) compared trials with distal action effects with trials without distal action effects. In Experiment 2, we tested the role of action effect contingency (i.e., a response was either always followed by the same or a random action effect. Non-contingent effects might hamper the termination of the event file, as the intended action goal is more likely not to be achieved compared to the contingent condition. Here, we (block-wise) compared trials in which the light bulbs reacted in a contingent way to the keypresses with trials where the connection between keypresses and light bulbs was non-contingent (i.e., random). Finally, to consider the unique methodological aspect of the long-lasting action effects (i.e., light bulbs staying on until the next keypress), we conducted Experiment 3. Here, trials with light bulbs staying on ("long-lasting") alternated with trials in which the light bulbs went out again shortly after the button was pressed ("short-lasting"). This condition was intended to serve as a comparison with the operating principle of conventional action effects in reaction time experiments.

## Experiment 1: Long-lasting action effect vs. no action effect

### Method

#### Participants

The sample size was based on the study by Frings et al. (2023), who also investigated the influence of action effects on S-R binding. In their study, 30 participants were tested to observe S-R binding effects with a typical effect size of *d* ~ 0.55 (α = 0.05, one-tailed testing, 1-β > 0.80). As Frings et al. (2023) did not find an effect for non-semantic distal action effects we doubled our sample to at least 60 participants. Accordingly, 64 students (to accommodate potential outliers) at Trier University were recruited via the universities online recruiting system. Three participants were excluded because of incomplete data or because they were heavy outliers (as regards reaction times, error rates, or number of finished trials, following the classification by Tukey, 1977, pp. 39–43). 61 participants (46 female; 56 right-handed) remained (*M* = 22.18 years, range 18 to 37). Participants received credits for their study participation.

#### Design

The design was a DRB-Paradigm (Frings et al., 2007), modified with action effects. Three within-participant factors were varied trial-wise: response relation (response repetition vs. response change), distractor relation (distractor repetition vs. distractor change), and long-lasting action effect occurrence (effect vs. no effect).

#### Apparatus and stimuli

The experiment was programmed in PsychoPy (Version 2022.2.4, Peirce et al., 2019) and run online via Pavlovia. Four displays (prime, prime effect, probe, and probe effect) were presented in each trial. All stimuli were presented on a grey (RGB_255:_ 128, 128, 128) background. In the prime display, a triplet of three letters (two identical distractor letters and one target letter, font size: 25 pixels) was presented with the distractor letters flanking the target letter. In both prime and probe displays, the target letter and the distractor letters were always displayed in white (RGB_255_: 255, 255, 255). The target letter could either be an “F” or a “J”, and the distractor letters could either be a “D” or a “K”. One lightbulb was shown above and one underneath the letter triplet (each 50 × 50 pixels; 45 pixels above/underneath the letter triplet). The lightbulb could be grey (i.e., switched off, RGB_255_: 128, 128, 128) or yellow (i.e., switched on, RGB_255_: 255, 192, 0).

#### Procedure

Participants were presented with written instructions on the screen. They were instructed to carry out the task as quickly and as correctly as possible. Participants were instructed to place the left index finger on the F key and the right index finger on the J key (to ensure equal distances between the hands and the body, they were instructed to place the keyboard horizontally so that the space bar was in front of the center of their body). Importantly, participants were informed in a separate instruction that the response keys were connected to the light bulbs and would activate or deactivate them. It was also pointed out that the light bulbs would not work in some trials. Participants then had to complete a training block consisting of 6 trials. During the training, participants received feedback ("correct"or"incorrect") after each given answer. Participants were free to repeat the training trials as many times as they wished. In the experimental blocks, only incorrect answers or answers given too late were commented on. The maximum time for the response in the probe display was 2000 ms.

In the experimental block, participants had to respond to the prime and the probe display. The task was to identify the letter shown in the middle by pressing the corresponding key (F key for an"F"and J key for a"J"^3^).

In the prime display, lightbulbs were always switched off. In the prime effect display, following the keypress, the letters disappeared, and the lightbulbs changed or did not change, depending on the long-lasting action effect occurrence condition (effect vs. no effect). In the effect condition, one lightbulb was switched on, corresponding to the pressed key. In the no effect condition, the lightbulbs remained switched off.

**Fig. 1:**
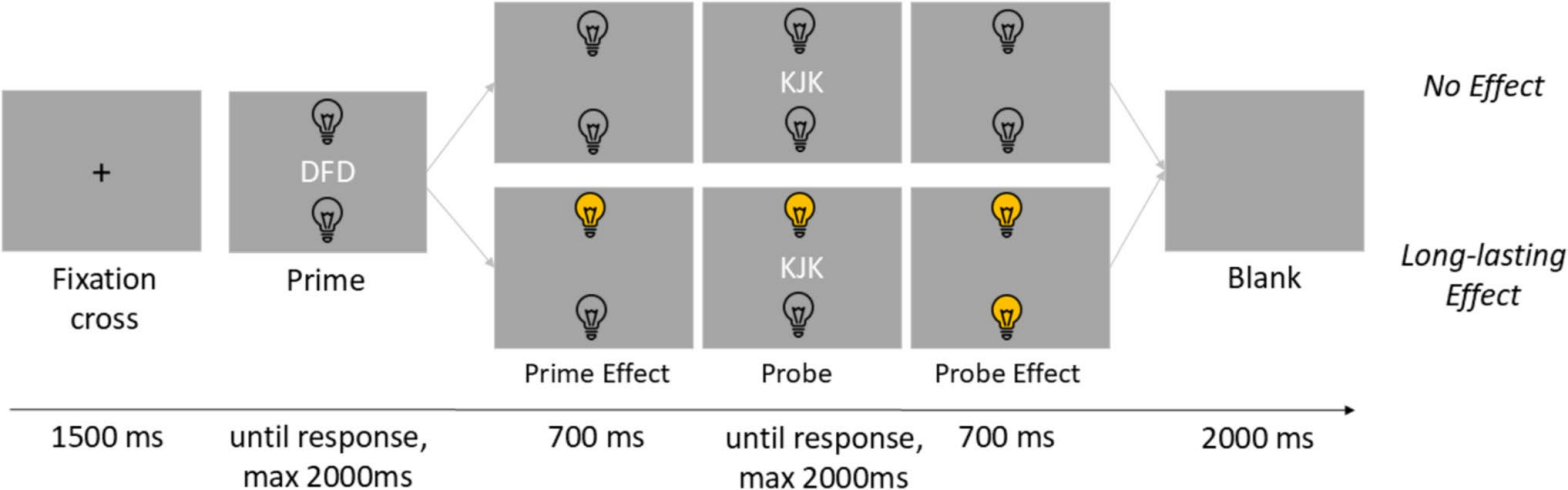
Experimental Procedure in Experiment 1. *Note*. Shown are trials with no action effect and long-lasting action effect. Stimuli are not drawn to scale

In the probe display, lightbulbs were either switched off (in the no effect condition) or the lightbulb switched on as a prime effect was still activated. In the probe effect display following the probe response, the letters disappeared, and (in the effect condition) the lightbulbs changed depending on the keypress (either the active lightbulb was switched off or the disabled lightbulb was switched on). At the end of the probe effect display, all stimuli disappeared. Therefore, independent of the condition, the lightbulbs appeared with the prime display and remained visible for the whole trial except the 2000 ms inter-trial interval (see Fig. 1). Response relation (repetition vs. change), distractor relation (repetition vs. change), and long-lasting action effect occurrence (effect vs. no effect) were counterbalanced across the experiment.

The experimental block consisted of 256 trials, so each letter was the probe-target 128 times. After every 20 th trial, there was a break, which the participants were allowed to finish themselves. Each single trial consisted of the same display sequence: A fixation cross was shown for 1500 ms. In the prime display, the target letter, distractor letters, and the lightbulbs appeared simultaneously. The letters lasted for a maximum of 2000 ms. The participants’ response ended the display and led to the prime effect display, which lasted for 700 ms. The probe display lasted for a maximum of 2000 ms, and the letters disappeared with the participants'response, leading to the probe effect display, which lasted 700 ms. The trial ended with a blank screen (inter-trial interval) for 2000 ms. The letters “F” and “J” occurred equally frequently as prime and as probe-targets. The letters “D” and “K” occurred equally frequently as prime and as probe distractors.

The response relation (response repetition vs. response change), the distractor relation (distractor response vs. distractor change), and the action effect (effect vs. no effect) were varied orthogonally. In the effect condition, the lightbulbs were switched on and off depending on the keypresses in the prime and the probe display (J keypress for the upper lightbulb and F keypress for the lower lightbulb). In the no effect condition, the lightbulbs remained switched off for the whole trial.

### Results

Data processing and analysis were performed with R (R Core Team, 2019; R version 4.2.2). The feature binding effects were calculated using this formula: [Performance _Response-Repetition/Distractor-Change Trials_ – Performance _Response-Repetition/Distractor-Repetition Trials_] – [Performance _Response-Change/Distractor-Change Trials_ – Performance _Response-Change/Distractor-Repetition Trials_]. This formula consists of two parts: First, the benefit of distractor repetition in response-repetition trials (full repetition) compared to distractor changes in response-repetition trials (partial repetition) is calculated. From these positive repetition benefits, the negative costs are then subtracted that emerge from distractor repetition in response-change trials (partial repetition) compared to distractor change in response change trials (full change). The total value should therefore be positive if feature bindings occur.^4^ Feature binding effects were compared with post-hoc *t*-tests. See Table 1 for descriptive data.

**Table 1:** Descriptive data for Experiment 1

Response Relation	Distractor Relation	Action Effect Condition	Reaction Times (ms)	SD (ms)	CI (ms)	Error Rates (%)	SD (%)	CI (%)
RC	DC	No Effect	489.91	17.14	4.39	4.06	4.12	1.06
RC	DC	Effect	486.12	23.49	6.02	3.79	4.42	1.13
RC	DR	No Effect	494.13	17.69	4.53	5.17	3.47	0.89
RC	DR	Effect	491.83	24.99	6.40	3.29	3.28	0.84
RR	DC	No Effect	482.87	24.26	6.21	5.90	4.90	1.25
RR	DC	Effect	484.97	16.87	4.32	6.28	4.26	1.09
RR	DR	No Effect	459.48	23.89	6.12	1.39	2.38	0.61
RR	DR	Effect	470.94	23.96	6.14	3.04	3.97	1.02

#### Data processing

Trials with incorrect responses (for either prime or probe) were excluded from reaction time analyses (13.44% of trials, including time-outs). In addition, another 0.03% of trials with probe RT below 200 ms and more than 1.5 interquartile ranges above the third quartile of each subject’s RT distribution were excluded (see Tukey, 1977). Thus, a total of 14.47% of all trials were excluded from reaction time analyses. For error rate analyses, probe error rates were used. Trials with prime error were excluded (0.14% of all probe error trials).

#### Reaction times

**Fig. 2:**
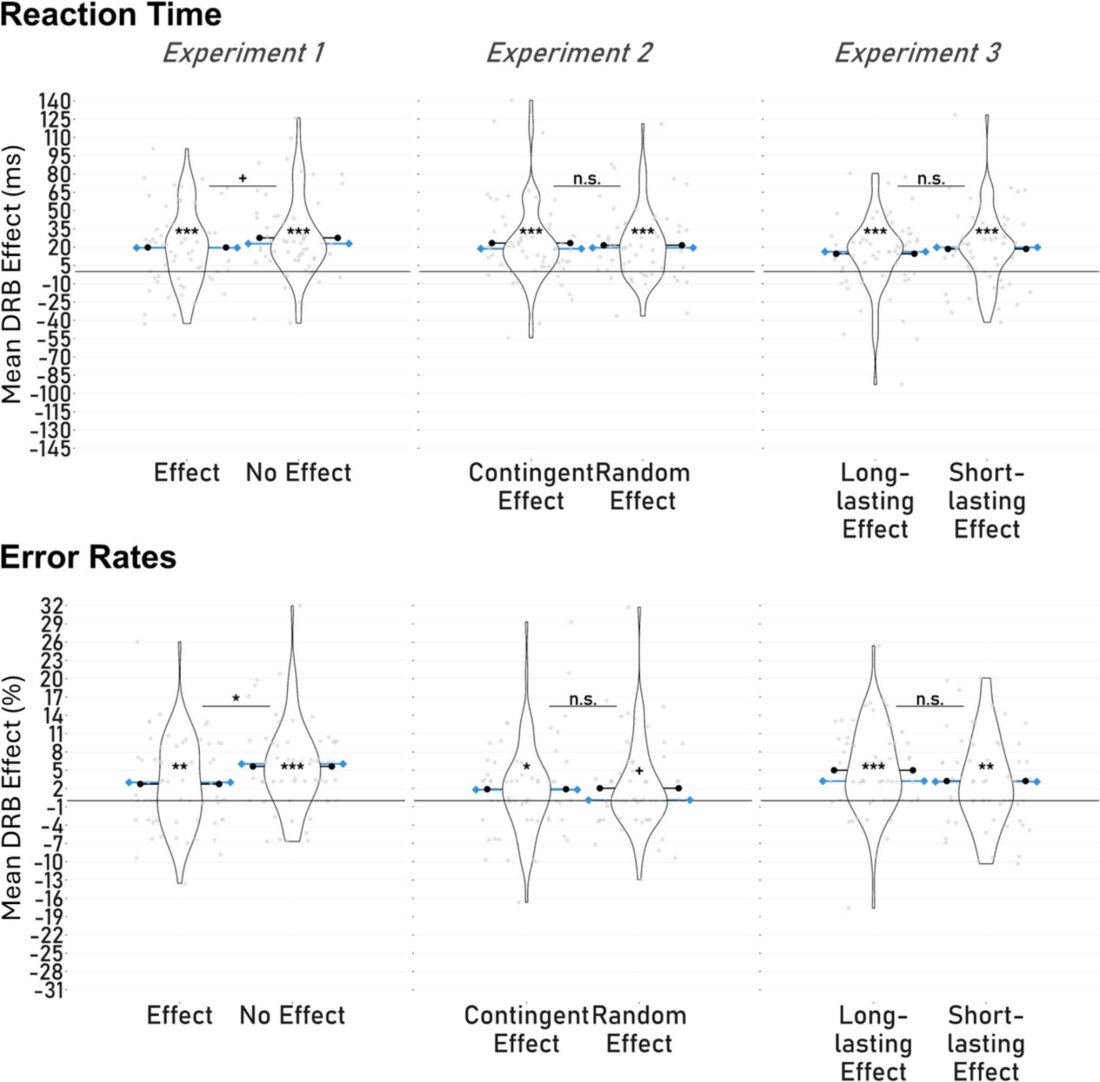
Results in Experiments 1—3*. Note*. The violin plots show the distribution of feature binding effects for both probe reaction times (ms) and probe error rates (%). In Experiment 1, long-lasting action effects alternated trial-wise with no action effects. In Experiment 2, contingent long-lasting action effects alternated block-wise with non-contingent/random long-lasting action effects. In Experiment 3, long-lasting action effects alternated trial-wise with short-lasting action effects. Grey dots mark individual data points. Means of the distributions are marked as black lines with dots, and medians as blue lines with diamonds. Stars and plus-signs within the violin plots indicate the significance level of the binding effects against zero (+ = *p* < 0.100, * = *p* < 0.050, ** = *p* < 0.010, *** = *p* < 0.001)

Both long-lasting action effect occurrence conditions produced significant feature binding effects. The feature binding effects in the effect condition (*M* = 20 ms, *SD* = 31 ms) were significantly different from zero, two-tailed* t(60) = 4.95, p* < 0.001, *d* = 0.84, as well as in the no effect condition (*M* = 28 ms, *SD* = 33 ms), two-tailed* t(60) = 6.56, p* < 0.001, *d* = 0.63. A paired *t*-test comparing the two action effect conditions revealed a marginally significant difference between the effect and the no effect condition: two-tailed *t(60) = 1.81, p* = 0.076, *d* = 0.23 (see Fig. 2). This suggested descriptively stronger feature binding effects for the no effect condition.^5^

A post-hoc power analysis was conducted (using G*Power; Faul et al., 2007) to assess the statistical power of the critical *t*-test comparison. Based on an observed effect size of *d* = 0.23, a significance level of α = 0.05, and a sample size of *n* = 61, the power was calculated to be 1-β = 0.42, indicating that there was a 42% chance of detecting an effect of this size. Furthermore, a sensitivity analysis was conducted to determine the smallest detectable effect size with a sample size of 61, a significance level of α = 0.05, and a desired power of 0.80: The analysis indicated that the experiment was powered to detect an effect size of at least *d* = 0.36.

#### Error rates

As in reaction times, both action effect conditions produced significant feature binding effects. The effect condition (*M* = 2.74%, *SD* = 7.21%) significantly differed from zero, two-tailed* t(60) = 2.98, p* = 0.004, *d* = 0.38, as did the no effect condition (*M* = 5.63%, *SD* = 7.22%), two-tailed* t(60) = 6.09, p* < 0.001, *d* = 0.78. Another paired *t*-test comparing the two action effect conditions in error rates revealed a significant difference between the effect and the no effect condition: two-tailed *t(60) = 2.31, p* = 0.024, *d* = 0.30. This indicated significantly stronger feature binding effects for the no effect condition.

A post-hoc power analysis regarding the critical *t*-test comparison (*d* = 0.23, α = 0.05, *n* = 61) led to a power of 1-β = 0.64, indicating a 64% chance of detecting an effect of this size (but see Zhang et al., 2019, for a critical view on the interpretability of post-hoc power analyses).

### Discussion

In Experiment 1, we found evidence for a modulation of the feature binding effect by the added action effect. Contrary to our assumption, the feature binding effect was more pronounced (especially in mean error rates) in trials with no action effect compared to trials with a long-lasting action effect. An explanation for this could be that the prime display in trials with action effect always differed from the probe display in terms of the lightbulbs (both lightbulbs switched off in the prime display and one lightbulb switched on in the probe display). That difference could have affected retrieval in the probe (cf., *encoding* specificity, Laub & Frings, 2020) and therefore caused smaller feature binding effects.

## Experiment 2: Action effect contingency

To test the assumption that *non-contingent* action effects hamper event file termination (and thus cause weaker feature binding effects), we conducted Experiment 2. Here, we compared trials with contingent effects with trials with non-contingent (i.e., random) effects.

### Method

#### Participants

The same sample size was targeted as in Experiment 1. Accordingly, 64 students at Trier University were recruited via the universities online recruiting system. Four participants were excluded because of incomplete data or because they were heavy outliers (following the classification by Tukey, 1977, pp. 39–43). 60 participants (50 female; 57 right-handed) remained (*M* = 22.27 years, range 18 to 33). Participants received course credits for their study participation.

#### Design

As in Experiment 1, the design was a modified DRB-Paradigm (Frings et al., 2007). Three within-participant factors were varied: response relation (response repetition vs. response change, trial-wise), distractor relation (distractor repetition vs. distractor change, trial-wise), and long-lasting action effect contingency (contingent vs. random, block-wise).

#### Apparatus and stimuli

The apparatus and stimuli used were identical to Experiment 1.

#### Procedure

The procedure was the same as in Experiment 1, besides the following exceptions.

First, participants worked on two experimental blocks (contingent block and random block). The block order was counterbalanced across the participants,^6^.^7^

Secondly, in both conditions (contingent and non-contingent) a long-lasting action effect occurred. Therefore, in the prime display, the lightbulbs were always switched off. In the prime effect display, after the response, the target and distractor letters disappeared, and one lightbulb was switched on. Which light bulb was activated according to which logic depended on the condition: In the contingent condition, one key was always connected to the same lightbulb. In the non-contingent (random) condition, the connection between the key and the lightbulb was always random – each key therefore led with a 50% probability to the activation of either the upper or the lower lamp.

In the probe display, the activated lightbulb was still switched on. Following the keypress, in the probe effect display, the letters disappeared, and the lightbulbs changed, again depending on the effect contingency condition: Either the active lightbulb was switched off or the disabled lightbulb was switched on.

**Fig. 3:**
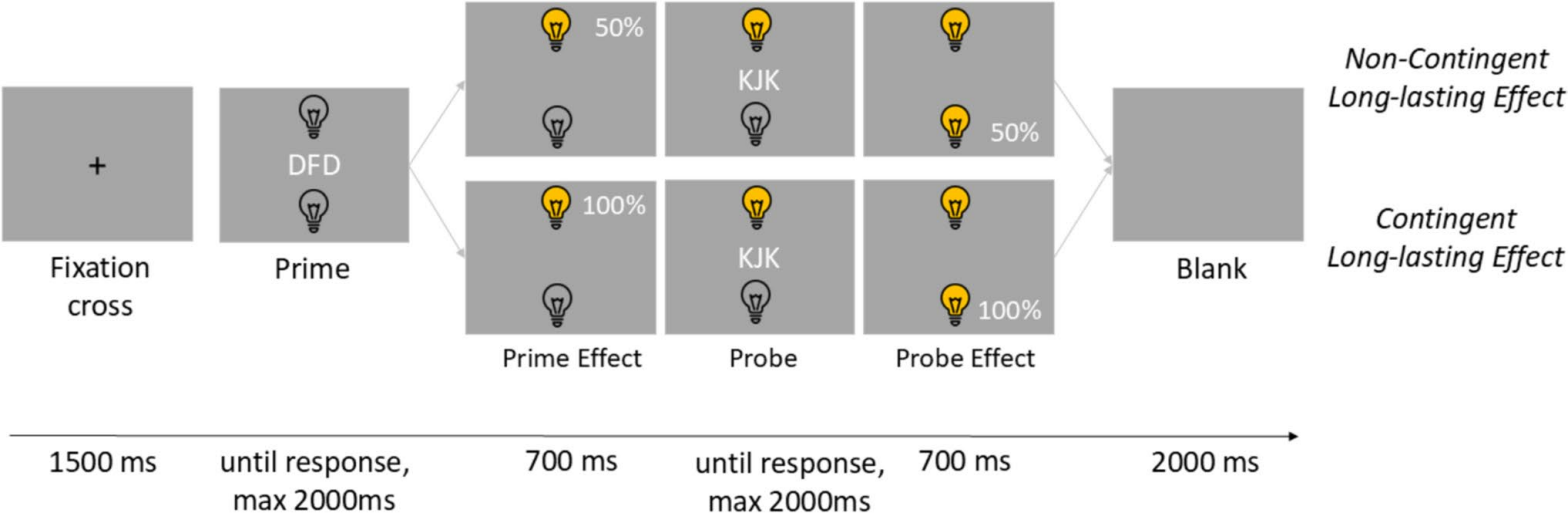
Experimental Procedure in Experiment 2. *Note*. Shown are trials with non-contingent and contingent lost-lasting action effect. In the non-contingent condition, one of the effects (i.e., upper or lower light bulb) followed the responses randomly: Depicted is a trial, in which the *upper* lightbulb was activated by pressing the F-key (50% probability for this combination), followed by activation of the *lower* lightbulb after pressing the J-key (50% probability). In the contingent condition, each of the two action effects always followed one of the two responses with 100% probability (i.e., F-key *always* activated the *upper* lightbulb and J-key *always* activated the *lower* lightbulb). Stimuli are not drawn to scale

The two experimental blocks each consisted of 128 trials, so each letter was the probe-target 64 times. Besides that, the sequence was the same as in Experiment 1 (see Fig. 3).

The response relation (response repetition vs. response change), the distractor relation (distractor response vs. distractor change), and the effect contingency (contingent vs. random) were varied orthogonally.

### Results

Data processing was analogous to Experiment 1. Analyses were further supplemented by an ANOVA to test for contingency and block order effects (see footnotes 6 and 7, and Table 2 for descriptive data).

**Table 2:** Descriptive data for Experiment 2

Response Relation	Distractor Relation	Action Effect Contingency	Reaction Times (ms)	SD (ms)	CI (ms)	Error Rates (%)	SD (%)	CI (%)
RC	DC	Non-Contingent	475.96	22.99	5.94	3.92	3.62	0.93
RC	DC	Contingent	470.30	23.59	6.09	4.46	4.05	1.05
RC	DR	Non-Contingent	481.32	24.27	6.27	2.98	3.13	0.81
RC	DR	Contingent	476.13	24.77	6.40	3.90	4.47	1.15
RR	DC	Non-Contingent	470.54	31.10	8.03	5.21	4.51	1.17
RR	DC	Contingent	466.63	24.71	6.38	5.41	4.21	1.09
RR	DR	Non-Contingent	452.61	21.13	5.46	2.38	3.60	0.93
RR	DR	Contingent	450.90	25.98	6.71	2.79	2.98	0.77

#### Data processing

Trials with incorrect responses (for either prime or probe) were excluded from reaction time analyses (15.6% of trials, including time-outs). In addition, another 1.3% of trials with probe RT below 200 ms and more than 1.5 interquartile ranges above the third quartile of each subject’s RT distribution were excluded (see Tukey, 1977). Thus, a total of 16.9% of all trials were excluded from reaction time analyses. For error rate analyses, probe error rates were used. Trials with prime error were excluded (0.11% of all probe error trials).

#### Reaction times

No main effect on reaction times was found for contingency (*F*(1, 59) = 1.57, *p* = 0.215, ${{\upeta }_{G}}^{2}$ < 0.01,${{\upeta }_{p}}^{2}$ = 0.03),^8^ indicating that it made no difference for the reaction times whether the currently processed block contained contingent or random action effects.

Furthermore, significant feature binding effects could be found in both action effect conditions. The contingent action effect condition (*M* = 22 ms, *SD* = 29 ms) was significantly different from zero, two-tailed *t(59) = 5.82, p* < 0.001, *d* = 0.75, as well as the random action effect condition (*M* = 23 ms, *SD* = 34 ms), two-tailed* t(59) = 5.32, p* < 0.001, *d* = 0.69. A paired* t*-test comparing the binding effects in both conditions revealed no significant difference between contingent and random action effects: two-tailed *t(59) = 0.35, p* = 0.731, *d* = 0.04 (see Fig. 2). This indicates that the contingency of distal action effects did not modulate feature binding effects.

A post-hoc power analysis of the critical *t*-test comparison (*d* = 0.04, α = 0.05, *n* = 60) led to a power of 1-β = 0.06, indicating a 6% chance to detect an effect of this size. A sensitivity analysis was conducted to determine the smallest detectable effect size (with a sample size of *n* = 60, a significance level of α = 0.05, and a desired power of 0.80). The analysis indicated that the experiment was powered to detect an effect size of *d* = 0.37 or larger.

#### Error rates

As for reaction times, no main effect on error rates was found for contingency: *F*(1, 59) = 2.21, *p* = 0.143, ${{\upeta }_{G}}^{2}$ < 0.01,${{\upeta }_{p}}^{2}$ = 0.04.^9^ In both action effect conditions, we observed significant respective marginally significant feature binding effects. The contingent action effect condition (*M* = 2.05%, *SD* = 7.16%) significantly differed from zero, two-tailed *t(59) = 2.21, p* = 0.031, *d* = 0.29, so did the random action effect condition marginally (*M* = 1.90%, *SD* = 7.62%), two-tailed* t(59) = 1.92, p* = 0.059, *d* = 0.25. A comparison between the two action effect conditions revealed no significant difference between contingent and random action effects: two-tailed *t(59) = 0.14, p* = 0.888, *d* = 0.02.

A post-hoc power analysis of the critical *t*-test comparison (*d* = 0.02, α = 0.05, *n* = 60) led to a power of 1-β = 0.05, indicating a 5% chance to detect an effect of this size.

### Discussion

Significant feature binding effects were observed in both contingency conditions, but no evidence was found for a modulation by the contingency manipulation. This indicates that action effect contingency did not influence action execution and feature bindings. Furthermore, this suggests that neither contingency enhanced nor random action effects hamper event file termination.

## Experiment 3: Long-lasting action effect vs. short-lasting action effect

In Experiment 3, we compared the action effect type used in Experiments 1 and 2 (i.e., light bulbs staying on until the next keypress or the end of the trial, “long-lasting”) to an action effect type more common in conventional reaction time experiments. Therefore, in the “short-lasting” action effect condition, the light bulbs went out again shortly after the button was pressed.^10^

### Method

#### Participants

The same sample size was targeted as in Experiments 1 and 2. Accordingly, 64 students at Trier University were recruited via the universities online recruiting system. Four participants were excluded because of being heavy outliers (following the classification by Tukey, 1977, pp. 39–43). 60 participants (45 female; 56 right-handed, 1 ambidextrous) remained (*M* = 23.54 years, range 19 to 34). Participants received credits for their study participation.

#### Design

The design was the same as in the effect condition in Experiment 1, but long-lasting action effects alternated with short-lasting action effects. Therefore, three within-participant factors were varied: response relation (response repetition vs. response change), distractor relation (distractor repetition vs. distractor change), and action effect type (long-lasting vs. short-lasting).

#### Apparatus and stimuli

The apparatus and stimuli used were identical to Experiment 1.

#### Procedure

The experimental setup was identical to Experiment 1, except that the long-lasting action effect was tested against a short-lasting action effect.

**Fig. 4:**
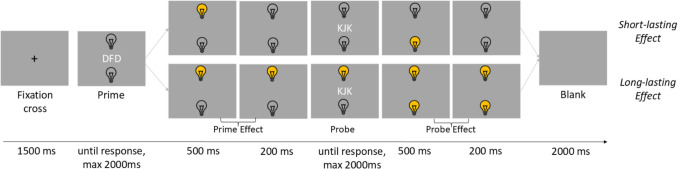
Experimental Procedure in Experiment 3. *Note*. Shown are trials with short-lasting action effects and long-lasting action effects. Note that for the short-lasting effect in both prime and probe effect displays, the activated lightbulb was always automatically switched off after 500 ms. Therefore, between switching off and probe onset/trial end, lightbulbs were switched off (but visible) for another 200 ms, to keep the effect display duration of 700 ms the same between all experiments and conditions. Stimuli are not drawn to scale

The experimental block consisted of 256 trials, so each letter was the probe-target 128 times. The response relation (response repetition vs. response change), the distractor relation (distractor response vs. distractor change), and the action effect type (long-lasting action effect vs. short-lasting action effect) were varied orthogonally. In the long-lasting action effect condition, the lightbulbs were switched on and off depending on the keypresses in the prime, and the probe display (J keypress for the upper lightbulb and F keypress for the lower lightbulb), remained switched on for the complete effect display and kept their condition for the probe display. In the short-lasting action effect condition, the lightbulbs were switched on depending on the keypresses in the prime and the probe display but were automatically switched off after the first 500 ms of the probe display had passed (see Fig. 4).

### Results

Data processing and analysis were performed analogously to Experiment 1. See Table 3 for descriptive data.

**Table 3:** Descriptive data for Experiment 3

Response Relation	Distractor Relation	Action Effect Type	Reaction Times (ms)	SD (ms)	CI (ms)	Error Rates (%)	SD (%)	CI (%)
RC	DC	long-lasting	469.37	19.92	5.19	3.57	3.52	0.92
RC	DC	short-lasting	468.68	22.42	5.84	3.03	3.28	0.85
RC	DR	long-lasting	471.64	17.15	4.47	4.67	3.80	0.99
RC	DR	short-lasting	471.64	20.41	5.32	3.45	3.28	0.85
RR	DC	long-lasting	448.80	18.17	4.74	5.64	4.00	1.04
RR	DC	short-lasting	460.64	18.67	4.87	5.84	4.35	1.13
RR	DR	long-lasting	436.48	18.29	4.77	1.73	3.16	0.82
RR	DR	short-lasting	445.09	21.86	5.70	3.03	3.55	0.93

#### Data processing

Trials with incorrect responses (for either prime or probe) were excluded from reaction time analyses (9.0% of trials, including time-outs). In addition, another 3.6% of trials with probe RT below 200 ms and more than 1.5 interquartile ranges above the third quartile of each subject’s RT distribution were excluded (see Tukey, 1977). Thus, a total of 12.6% of all trials were excluded from reaction time analyses. For error rate analyses, probe error rates were used. Trials with prime error were excluded (0.07% of all probe error trials).

#### Reaction times

We observed significant feature binding effects in both action effect conditions. The short-lasting action effect (*M* = 16 ms, *SD* = 37 ms) was significantly different from zero, two-tailed* t(59) = 3.33, p* = 0.001, *d* = 0.43, was well was the long-lasting action effect (*M* = 15 ms, *SD* = 31 ms), two-tailed* t(59) = 3.78, p* < 0.001, *d* = 0.49. However, again we found no significant difference in feature binding effects between the short-lasting and the long-lasting action effect condition: two-tailed *t(59) = 0.15, p* = 0.878, *d* = 0.02 (see Fig. 2). This indicates that the type of distal action effect – long-lasting or short-lasting – did not influence feature binding effects.

A post-hoc power analysis of the critical *t*-test comparison (*d* = 0.02, α = 0.05, *n* = 60) led to a power of 1-β = 0.05, indicating a 5% chance to detect an effect of this size. A sensitivity analysis was conducted to determine the smallest detectable effect size (with a sample size of *n* = 60, a significance level of α = 0.05, and a desired power of 0.80). The analysis indicated that the experiment was powered to detect an effect size of at least *d* = 0.37.

#### Error rates

As in reaction times, we found significant feature binding effects in both action effect conditions. The short-lasting action effect (*M* = 3.28%, *SD* = 7.31%) significantly differed from zero, two-tailed* t(59) = 3.47, p* = 0.001, *d* = 0.45, as did the long-lasting action effect (*M* = 4.98%, *SD* = 7.15%), two-tailed* t(59) = 5.40, p* < 0.001, *d* = 0.70. No significant difference emerged between the short-lasting action effect and the long-lasting action effect condition: two-tailed *t(59) = 1.32, p* = 0.193, *d* = 0.17.

A post-hoc power analysis of the critical *t*-test comparison (*d* = 0.17, α = 0.05, *n* = 60) led to a power of 1-β = 0.25, indicating a 25% chance to detect an effect of this size.

### Discussion

Experiment 3 showed no difference between the (closer to everyday use) long-lasting and the (more conventional) short-lasting action effect. This suggests that our findings from Experiments 1 and 2 can be extended to paradigms using more conventional action effects.

## General discussion

In the present study, we investigated how a semantically enriched and overlearned distal action effect (a lightbulb) would affect stimulus–response bindings and, as a mediator in this process, event file termination. For this purpose, we used a “long-lasting” action effect (Experiments 1 and 2) that was supposed to reflect the everyday use of light switches where lights remain switched on until the switch is pressed again. We compared trials causing this action effect with trials with no effect (Experiment 1) and with trials in which the effect would not occur contingently on always the same corresponding key, but randomly (Experiment 2). In Experiment 3, we introduced another type of action effect (“short-lasting”, i.e., lightbulbs went off automatically after a short period of time) that was more similar to the action effects used in conventional reaction time experiments. Here, we directly compared the “long-lasting” with the “short-lasting” action effect.

Event file termination is assumed to be a process necessary for retrieval (Frings et al., 2023). It is known that the perceptual stream is segmented into episodes (e.g., Kurby & Zacks, 2008; Zacks & Swallow, 2007) and that these episodes are retrievable (e.g., Zheng et al., 2022). Thus, there must be a component in an action episode that marks the end of this episode (i.e., event file termination). This component might depend on the action mode (cf., Herwig & Waszak, 2012): An intention-based (in contrast to stimulus-based) mode should lead to an action being carried out in terms of the intended effect (and less so as a mere reaction to a stimulus), with the action being represented by the anticipated change in the environment and not as a stimulus-triggered reaction (cf. Kunde et al., 2007). This should influence what is used as a marker for achieving an action goal: Either the reaction or the effect. Consequently, the event file should be terminated either by the reaction or by the perception of the effect.

Frings et al. (2023) already tried to tackle event file termination by using distal action effects and found no significant modulations. Critically, they used arbitrary action effects. By using a salient and semantically meaningful action effect (i.e., the"long-lasting"lightbulbs), we expected a shift from a stimulus-based to an intention-based action mode (cf., Herwig & Waszak, 2012) since the link between the response and our action effect (switching light bulbs on and off) is overlearned in everyday life. Thus, in our Experiments, we expected observable modulations of the feature binding effect by the different manipulations of the additional distal action effect.

Importantly, due to the 1-to-1 mapping that was used in all paradigms, we cannot clearly attribute our effects purely to *stimulus–response* binding since it cannot be ruled out that only the target stimuli were bound to the distractor stimuli (*stimulus-stimulus* binding) and not the responses. Due to this limitation, we do not interpret our results in terms of S–S or S-R binding, but more generally for *feature binding* effects to account for both possibilities. In this context, reference should be made to Frings et al. (2007): Here, distractor-response binding effects were compared depending on whether the target stimulus was repeated or not. It was found that the distractor relation also had a clear influence on the response in the case of target repetition. Thus, it can be assumed, at least for our study, that the observed feature binding effects show a significant proportion of S-R binding (and not only S–S binding). For further discussion, however, this matter should still be considered, since for S–S bindings a different relevance of (distal) action effects should be assumed than for S-R bindings (cf. Moeller et al., 2019).

However, while stable feature binding effects were present in all conditions of all 3 experiments, only Experiment 1 showed a modulation by the action effect (and in the opposite direction to what we expected): Trials without the"long-lasting"action effect showed significantly higher feature binding effects (especially in mean error rates) than trials with the action effect. The remaining General Discussion thus critically reflects the non-significant findings. Yet, it must be acknowledged that although we used rather large sample sizes for these kinds of experiments, post-hoc power analyses showed that we would not have detected small effect sizes. Thus, there might be principally an impact of distal action effects but under the conditions we used, we can for sure say this impact is rather small (cf., Lakens, 2022).

Additional distal action effects thus did not show a clear modulation of the feature binding effects in our study. Various explanations can be put forward for these rather ambiguous findings. However, these can neither be clearly confirmed nor rejected based on the present data. First, it could be due to a contextual issue caused by the “long-lasting” action effect, namely encoding specificity (Laub & Frings, 2020). According to this principle, a change between the encoding context and the retrieval context weakens retrieval and therefore the resulting feature binding effects. This was the case in Experiment 1: In trials with the action effect, the probe display always differed from the prime display in terms of the lightbulbs – one lightbulb was always switched on. In trials without action effect (the lightbulbs remained switched off), prime and probe displays were always the same (except for the stimulus and distractor letters). However, since in Experiment 3 the same change in encoding and retrieval context did not significantly modulate feature bindings, this explanation seems to be unlikely.

Furthermore, a rather technical issue arises from the long-lasting action effect. With this type of effect, a response repetition always caused an action effect change: That is, because by pressing the key in the prime, a lightbulb was switched on, and by responding with the same keypress in the probe, the lightbulb was switched off. It therefore depended on how the action was represented—namely via the stimulus or via the effect—whether a trial was rather processed as a *response-repetition* or an *effect-change* trial. This type of representation should depend on which action focus/mode was activated (cf. Kunde et al., 2007; Herwig & Waszak, 2012). Interestingly, this might affect the strength of the feature binding effect in Experiment 1: Response change distractor repetition trials would only be processed as *partial repetition* trials (leading to partial repetition costs and therefore feature binding effects) if the action execution was focused on action triggers (i.e., target stimuli). However, when the focus was on the intended action (i.e., the distal action effect), response *repetition* distractor repetition trials would also be processed as effect *change* distractor repetition trials. A full repetition trial would therefore be processed rather as a partial repetition trial (and a full change trial in the same way), leading to a reduction in feature binding effects. Assuming that our stimuli were indeed both everyday and semantic enough to switch the focus of action to cause a change in the environment, our data might give us a hint that this actually happened in Experiment 1.

In Experiments 2 and 3, however, no modulation of feature binding effects by the contingency of the additional distal action effect or by the type of action effect was observed. This outcome, in combination with the assumption that the pattern found in Experiment 1 can be attributed to rather methodological factors, suggests that additional distal action effects (i.e., meaningful changes in the environment that follow an action) did not influence feature bindings. Therefore, it might be that our semantic effects (or our paradigm per se) were not yet suited to induce an intention-based mode of action. Apart from the effects observed in Experiment 1, which suggest the *processing* of distal effects, we have little evidence of how the action effects were actually handled. It is possible, though, that in forced-choice (S-R) binding paradigms, participants generally do not use any mode other than a stimulus-based one – Many paradigms that successfully modulate bindings through action effects use *free-choice* tasks (e.g., Dutzi & Hommel, 2009).

Regarding event file termination, we found no evidence that additional distal action effects, as we have operationalized them in our feature binding paradigm, played any role. Stimulus-based tasks might thus use other markers to specify episodic boundaries. Possibly, event file termination depends on a perception that occurs before additional distal effects appear: In our case, that would be the disappearance of the letters as well as the proximal effects of the keystroke. Interestingly, the disappearance of the letters (comparable to a screen turning blank after the response) was ruled out in Frings et al. (2023), who used an S-R binding paradigm, where no modulation of S-R binding effects through this kind of distal action effect was found. Given this, what remains to be used as a marker for the end of the action episode might be the *proximal* effect: This action effect involves the proprioceptive and tactile feedback during the keypress and is therefore inseparable from the response. In fact, that underlines basic ideomotor theorizing according to which body-related perceptions are sufficient for *basic* action control (Pfister, 2019). This also corresponds to the results of other S-R binding tasks including a distal action effect, according to which the response is only retrieved by stimuli that are responded to and not by effect stimuli (Moeller et al., 2019). However, it remains a matter of debate how event file termination is influenced by distal and/or proximal action effects and, based on this, how event file termination actually affects feature binding – whether/when termination is necessary for feature binding, which features (stimulus or response features) are influenced by it and which situational and intentional factors modulate all of this.

Research on event file processes should therefore focus even more on the distinction between distal and proximal action effects, as different paradigms may influence how and when these effect types affect event file management. Although anticipated distal effects can undoubtedly be highly relevant for action execution and planning, their relation to and influence on other stimulus and response features within an event file remains rather unclear.

### Conclusion

In conclusion, we found no clear evidence that adding semantically meaningful distal action effects modulated feature binding effects. It has to be acknowledged though that our action effects were still somewhat artificial although previous studies could demonstrate the impact of these close-to-every-day-life action effects (Hommel, 1993). Taken together there is cumulating evidence that additional distal action effects do not affect feature binding – at least when paradigms are used in which stimulus–response associations are sufficient to meet the requirements of the specific task. In turn, event file termination and retrieval do not necessarily seem to depend on the occurrence or contingency of additional distal action effects. This changes the way we interpret dynamic event file management in typical feature binding tasks: It becomes clear that classic *S-R-based* paradigms are not functional to investigate the influence of distal effects on event file management due to the stimulus-based mode required there.

## Authorships contribution

**Nicolas D. Münster**: Conceptualization, Investigation, Methodology, Software, Formal analysis, Visualization, Writing—original draft, Writing – review & editing, Project administration.

**Philip Schmalbrock**: Software, Writing—original draft, Writing – review & editing.

**Christian Beste**: Writing – review & editing, Funding acquisition.

**Alexander Münchau**: Writing – review & editing.

**Christian Frings**: Conceptualization, Resources, Writing—original draft, Writing – review & editing, Funding acquisition, Supervision.

## Supplementary Information

Below is the link to the electronic supplementary material.

Supplementary file1 (DOCX 546 KB)

## Data Availability

The data for all experiments are available at PsychArchives under 10.23668/psycharchives.14232. Materials are not available, as they are described in detail in the text and can therefore be easily reproduced.

## Footnotes

1 By using this formula: [Performance _Feature A-Repetition/Feature B-Change Trials_ – Performance _Feature A-Repetition/Feature B-Repetition Trials_] – [Performance _Feature A-Change/Feature B-Change Trials_ – Performance _Feature A-Change/Feature B-Repetition Trials_] (see Frings et al., 2007). Performance refers to probe reaction times and probe error rates. Features A and B can be response (R) or stimulus (S) features, e.g., in an S-R binding task.

2 In this task, a cued response (R1) is given with the appearance of a first response display. The response is bound to the stimuli (S1) of this display. In a subsequent second response display, the response (R2) is given depending on the presented stimuli (S2). In this way, stimulus and response repetition/change can be varied independently from each other.

3 Since due to the 1-to-1 mapping target stimuli and responses cannot be separated from each other, some problems arise with regard to the differentiation between S–S and S-R binding (see the General Discussion on this topic). Our results are thus interpreted in terms of *feature bindings* (which probably combine proportions of S-R and S–S bindings; cf. Frings et al., 2007).

4 Note that calculating a feature binding effect simplifies the analysis processes because simple *t*-tests can be calculated, instead of ANOVA + post-hoc tests. The feature binding effect essentially breaks down the two-way interaction response relation × distractor relation (that signifies feature binding effects and yields four different values) into a single value. In Experiment 1, for example, we calculated one binding effect for each of the two action effect conditions. Comparing these two binding effects via *t*-tests yields the same results as an ANOVA looking at the three-way interaction response relation × distractor relation × condition. For ANOVA outputs and additional result plots of all 3 experiments see the Supplementary.

5 See Supplementary 5 for descriptive data of probe reaction times depending on the action effect order in consecutive trials.

6 For error rates, the block order did not interact with the feature binding effects: 3-way interaction between response relation, distractor relation and block order: *F*(1, 58) = 0.02, *p* =.892, ${{\upeta }_{G}}^{2}$ < 0.01,${{\upeta }_{p}}^{2}$ < 0.01, nor with the modulation of feature binding effects by contingency: 4-way interaction between response relation, distractor relation, contingency and block order:* F*(1, 58) = 0.01, *p* =.919, ${{\upeta }_{G}}^{2}$ < 0.01,${{\upeta }_{p}}^{2}$ < 0.01 (see Supplementary 2B for the full ANOVA output).

7 For reaction times, the block order did not interact with the feature binding effects: 3-way interaction between response relation, distractor relation and block order: *F*(1, 58) = 0.10, *p* =.761, ${{\upeta }_{G}}^{2}$ < 0.01,${{\upeta }_{p}}^{2}$ < 0.01, nor with the modulation of feature binding effects by contingency: 4-way interaction between response relation, distractor relation, contingency and block order:* F*(1, 58) = 1.59, *p* =.212, ${{\upeta }_{G}}^{2}$ < 0.01,${{\upeta }_{p}}^{2}$ = 0.03 (see Supplementary 2A for the full ANOVA output).

8 In a 2 (response relation) × 2 (distractor relation) × 2 (contingency) ANOVA on probe reaction times (using ‘ezAnova’ function of ‘ez’, Lawrence, 2016).

9 In a 2 (response relation) × 2 (distractor relation) × 2 (contingency) ANOVA on probe error rates.

10 See Supplementary 4 for an additional experiment that tested the short-lasting action effect against no action effect. Here, no effect of the action effect condition on S-R binding effects occurred.

## Code availability

Analysis scripts and codes are not available online (but can be provided on request), as all analysis steps are described in detail in the text and can therefore be easily reproduced.

## Declarations

### Open practices statement

The data for all experiments are available at PsychArchives under 10.23668/psycharchives.14232. None of the experiments was preregistered.

### Authors Notes

The Deutsche Forschungsgemeinschaft supported the research reported in this article (FOR 2698, FR2133/18–1). The content of this manuscript has not been disseminated before.
