# Supplementary material for: Adding meaningful distal action effects in feature binding
Source: Atten Percept Psychophys. 2025 May 20;87(5):1650–64. doi: 10.3758/s13414-025-03092-9 (PMC12204946; doi:10.3758/s13414-025-03092-9)
Supplement: Supplementary file 1 — Supplementary file1 (DOCX 546 KB) [file 13414_2025_3092_MOESM1_ESM.docx]

**Supplementary 1A**

*Exp1: ANOVA output (RT)*

| Predictor | *df_Num_* | *df_Den_* | *F* | *p* | η^2^_p_ |
| --- | --- | --- | --- | --- | --- |
| (Intercept) | 1 | 60 | 2399.46 | .000 | .98 |
| Response Relation | 1 | 60 | 20.86 | .000 | .26 |
| Distractor Relation | 1 | 60 | 22.43 | .000 | .27 |
| Action Effect Condition | 1 | 60 | 0.64 | .428 | .01 |
| Response Relation x Distractor Relation | 1 | 60 | 46.34 | .000 | .44 |
| Response Relation x Action Effect Condition | 1 | 60 | 11.81 | .001 | .16 |
| Distractor Relation x Action Effect Condition | 1 | 60 | 5.76 | .020 | .09 |
| Response Relation x Distractor Relation x Action Effect Condition | 1 | 60 | 3.27 | .076 | .05 |

*Note.* *df_Num_* indicates degrees of freedom numerator. *df_Den_* indicates degrees of freedom denominator. η^2^_p_ indicates partial eta-squared.

*Lineplot showing the reaction times for all conditions in Experiment 1.*
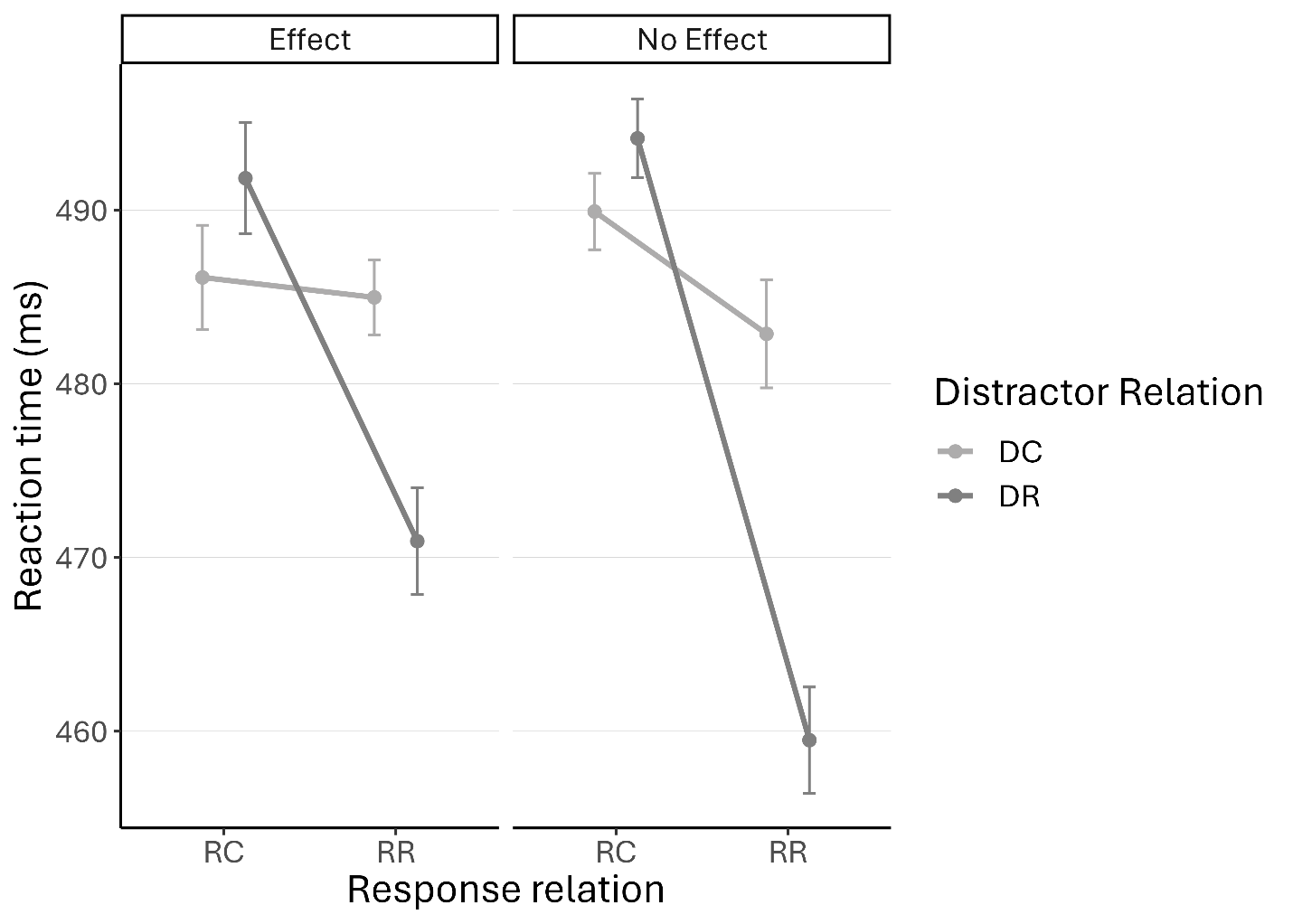


*Note*. Error bars depict the standard error of the mean.

**Supplementary 1B**

*Exp1: ANOVA output (Error rates)*

| Predictor | *df_Num_* | *df_Den_* | *F* | *p* | η^2^_p_ |
| --- | --- | --- | --- | --- | --- |
| (Intercept) | 1 | 60 | 167.43 | .000 | .74 |
| Response Relation | 1 | 60 | 0.03 | .868 | .00 |
| Distractor Relation | 1 | 60 | 33.83 | .000 | .36 |
| Action Effect Condition | 1 | 60 | 0.01 | .925 | .00 |
| Response Relation x Distractor Relation | 1 | 60 | 37.75 | .000 | .39 |
| Response Relation x Action Effect Condition | 1 | 60 | 9.05 | .004 | .13 |
| Distractor Relation x Action Effect Condition | 1 | 60 | 0.06 | .803 | .00 |
| Response Relation x Distractor Relation x Action Effect Condition | 1 | 60 | 5.34 | .024 | .08 |

*Note.* *df_Num_* indicates degrees of freedom numerator. *df_Den_* indicates degrees of freedom denominator. η^2^_p_ indicates partial eta-squared.

*Lineplot showing the error rates for all conditions in Experiment 1.*

**
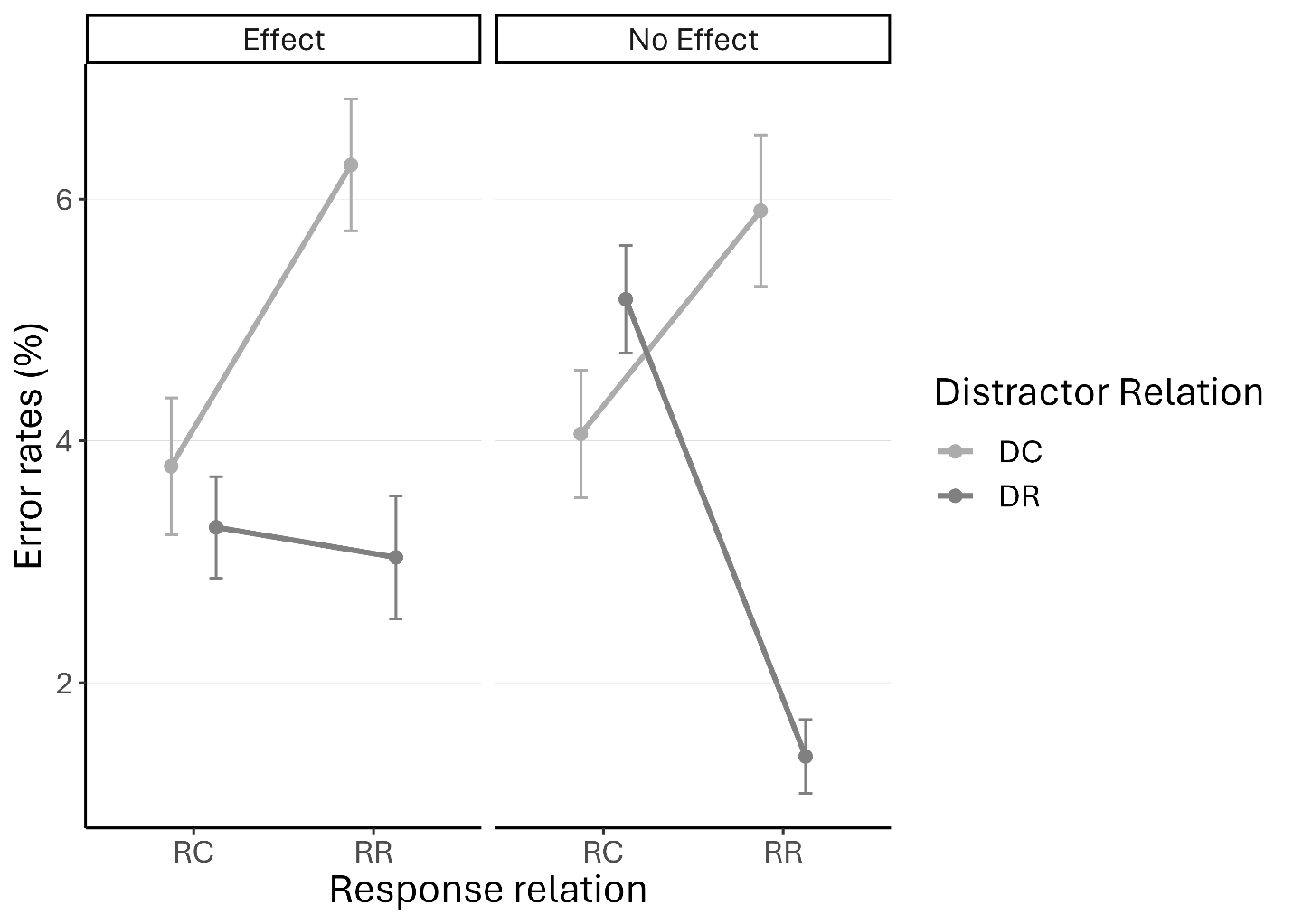
**

*Note*. Error bars depict the standard error of the mean.

**Supplementary 2A**

*Exp2: ANOVA output (RT)*

| Predictor | *df_Num_* | *df_Den_* | *F* | *p* | η^2^_p_ |
| --- | --- | --- | --- | --- | --- |
| (Intercept) | 1 | 59 | 3586.31 | .000 | .98 |
| Response Relation | 1 | 59 | 17.07 | .000 | .22 |
| Distractor Relation | 1 | 59 | 10.34 | .002 | .15 |
| Action Effect Contingency | 1 | 59 | 1.57 | .215 | .03 |
| Response Relation x Distractor Relation | 1 | 59 | 49.39 | .000 | .46 |
| Response Relation x Action Effect Contingency | 1 | 59 | 0.67 | .417 | .01 |
| Distractor Relation x Action Effect Contingency | 1 | 59 | 0.34 | .564 | .01 |
| Response Relation x Distractor Relation x Action Effect Contingency | 1 | 59 | 0.12 | .731 | .00 |

*Note.* *df_Num_* indicates degrees of freedom numerator. *df_Den_* indicates degrees of freedom denominator. η^2^_p_ indicates partial eta-squared.

*Lineplot showing the reaction times for all conditions in Experiment 2.*


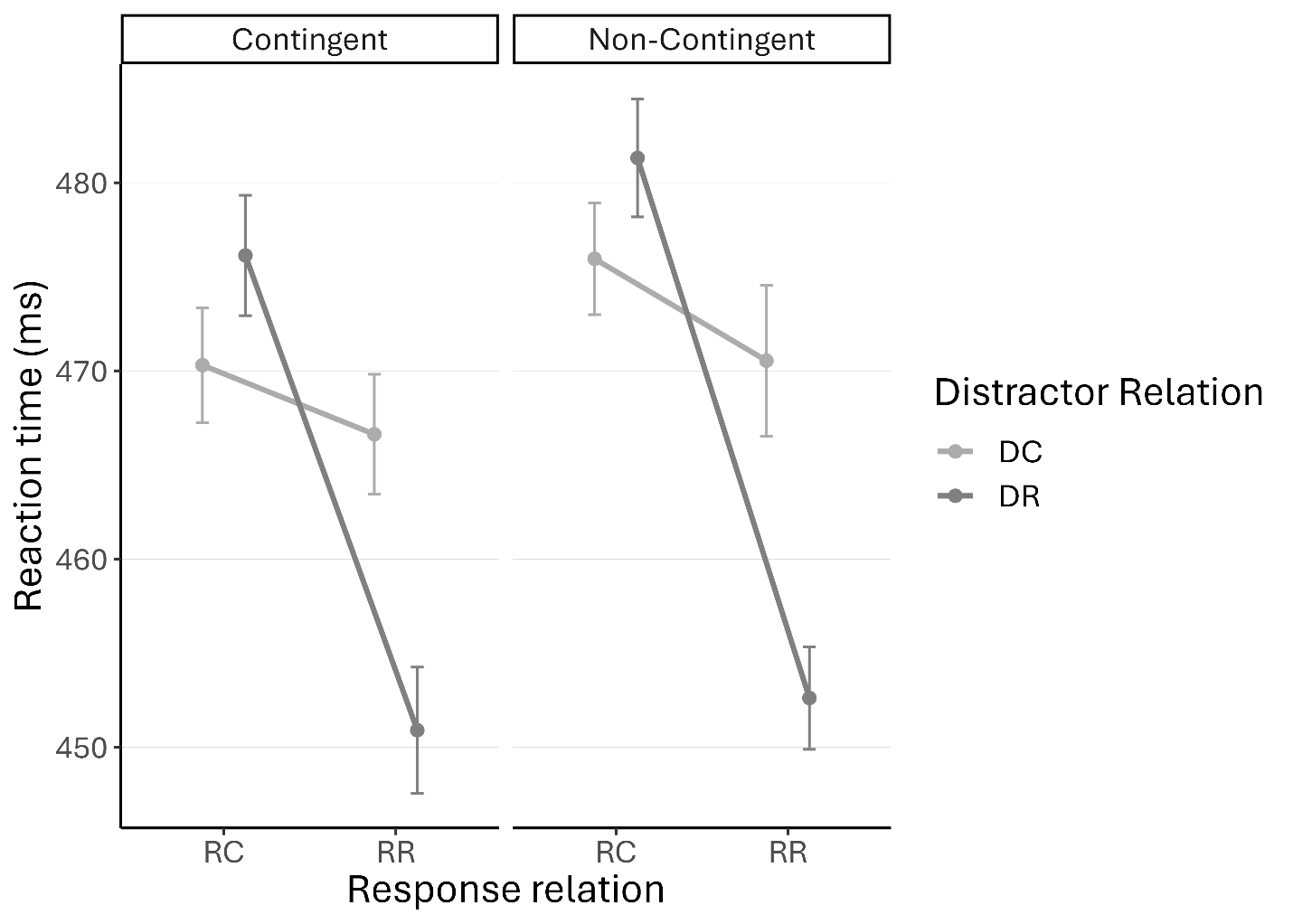


*Note*. Error bars depict the standard error of the mean.

**Supplementary 2B**

*Exp2: ANOVA output (Error rates)*

| Predictor | *df_Num_* | *df_Den_* | *F* | *p* | η^2^_p_ |
| --- | --- | --- | --- | --- | --- |
| (Intercept) | 1 | 59 | 140.05 | .000 | .70 |
| Response Relation | 1 | 59 | 0.08 | .772 | .00 |
| Distractor Relation | 1 | 59 | 30.33 | .000 | .34 |
| Action Effect Contingency | 1 | 59 | 2.21 | .143 | .04 |
| Response Relation x Distractor Relation | 1 | 59 | 6.37 | .014 | .10 |
| Response Relation x Action Effect Contingency | 1 | 59 | 0.35 | .555 | .01 |
| Distractor Relation x Action Effect Contingency | 1 | 59 | 0.23 | .632 | .00 |
| Response Relation x Distractor Relation x Action Effect Contingency | 1 | 59 | 0.02 | .888 | .00 |

*Note.* *df_Num_* indicates degrees of freedom numerator. *df_Den_* indicates degrees of freedom denominator. η^2^_p_ indicates partial eta-squared.

*Lineplot showing the error rates for all conditions in Experiment* **
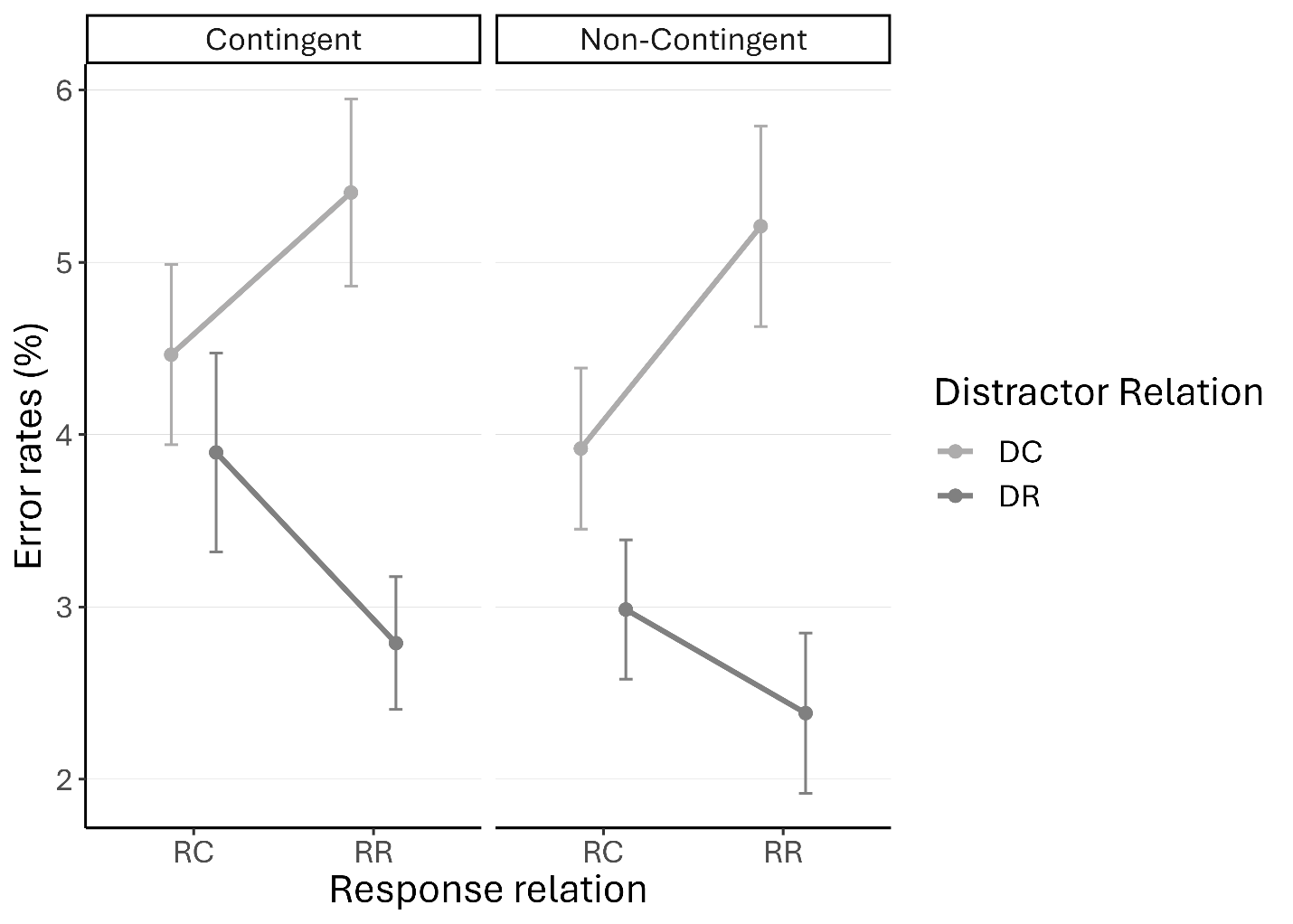
**

*Note*. Error bars depict the standard error of the mean.

**Supplementary 3A**

*Exp3: ANOVA output (RT)*

| Predictor | *df_Num_* | *df_Den_* | *F* | *p* | η^2^_p_ |
| --- | --- | --- | --- | --- | --- |
| (Intercept) | 1 | 58 | 5519.72 | .000 | .99 |
| Response Relation | 1 | 58 | 42.23 | .000 | .42 |
| Distractor Relation | 1 | 58 | 19.38 | .000 | .25 |
| Action Effect Type | 1 | 58 | 11.23 | .001 | .16 |
| Response Relation x Distractor Relation | 1 | 58 | 28.54 | .000 | .33 |
| Response Relation x Action Effect Type | 1 | 58 | 13.27 | .001 | .19 |
| Distractor Relation x Action Effect Type | 1 | 58 | 0.37 | .546 | .01 |
| Response Relation x Distractor Relation x Action Effect Type | 1 | 58 | 0.64 | .426 | .01 |

*Note.* *df_Num_* indicates degrees of freedom numerator. *df_Den_* indicates degrees of freedom denominator. η^2^_p_ indicates partial eta-squared.

*Lineplot showing the reaction times for all conditions in Experiment 3.*


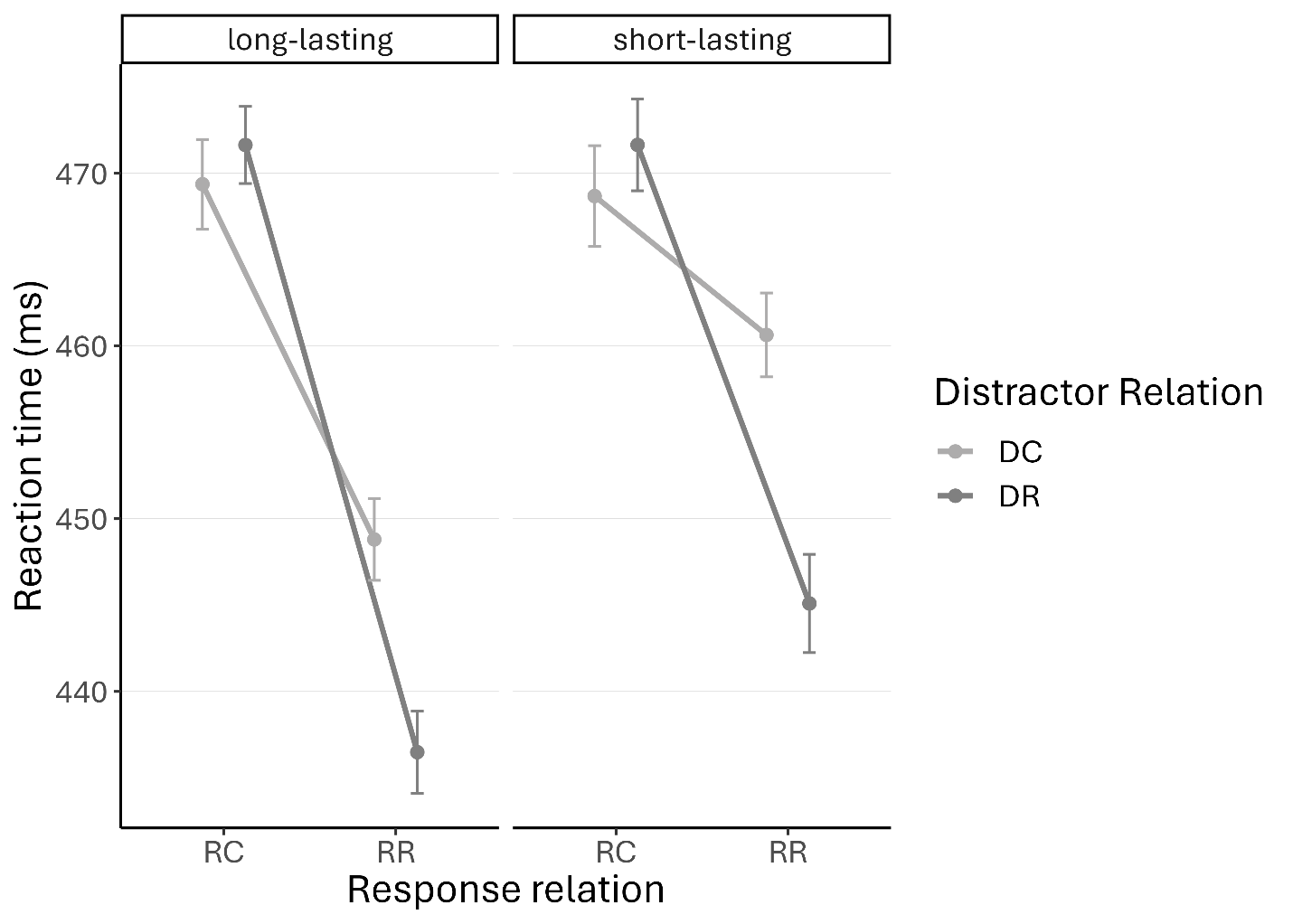

*Note*. Error bars depict the standard error of the mean.

**Supplementary 3B**

*Exp3: ANOVA output (Error rates)*

| Predictor | *df_Num_* | *df_Den_* | *F* | *p* | η^2^_p_ |
| --- | --- | --- | --- | --- | --- |
| (Intercept) | 1 | 58 | 170.62 | .000 | .75 |
| Response Relation | 1 | 58 | 0.94 | .337 | .02 |
| Distractor Relation | 1 | 58 | 13.84 | .000 | .19 |
| Action Effect Type | 1 | 58 | 0.04 | .848 | .00 |
| Response Relation x Distractor Relation | 1 | 58 | 36.09 | .000 | .38 |
| Response Relation x Action Effect Type | 1 | 58 | 7.11 | .010 | .11 |
| Distractor Relation x Action Effect Type | 1 | 58 | 0.14 | .713 | .00 |
| Response Relation x Distractor Relation x Action Effect Type | 1 | 58 | 1.83 | .181 | .03 |

*Note.* *df_Num_* indicates degrees of freedom numerator. *df_Den_* indicates degrees of freedom denominator. η^2^_p_ indicates partial eta-squared.

*Lineplot showing the error rates for all conditions in Experiment 3.*
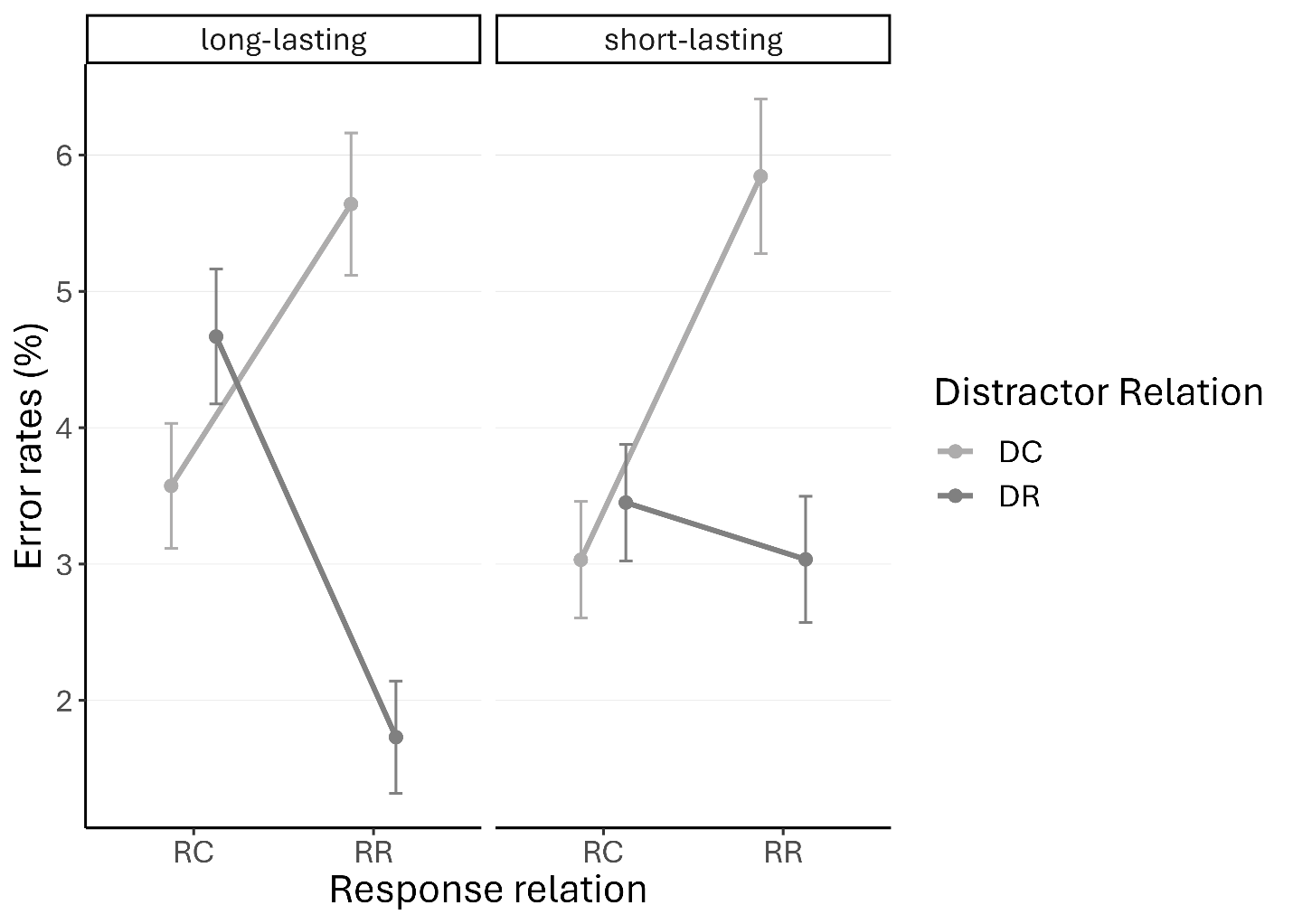


*Note*. Error bars depict the standard error of the mean.

**Supplementary 4**

*Results of an additional experiment testing short-lasting action effects against no action effects.*

64 participants took part in this experiment (*Exp4*). Two participants were excluded due to the criteria defined in the main text. 62 participants remained (41 female; 56 right-handed, *M* = 22.94 years, range 19 to 34). Stimulus material and procedure were the same as in Experiment 1, with one exception: long-lasting action effects were replaced with short-lasting action effects.

Significant S-R binding effects emerged for both effect conditions in both reaction times and error rates (against zero: reaction times: effect: t(61) = 3.17, *p* = .002, *d* = 0.40; no effect: t(61) = 3.50, *p* = .001, *d* = 0.44; error rates: effect: t(61) = 4.37, *p* < .001, *d* = 0.56; no effect: t(61) = 5.66, *p* < .001, *d* = 0.72). No difference were found in the observed S-R binding effects between both action effect conditions, reaction times: t(61) = 0.53, *p* = .596, *d* = 0.07; error rates: t(61) = 0.78, *p* = .439, *d* = 0.10.

Results demonstrate that the presence of a short-lasting effect did not affect S-R binding effects.

**Supplementary 4A**

*Experimental Procedure in additional Experiment 4: Short-lasting effect versus no effect.*

**
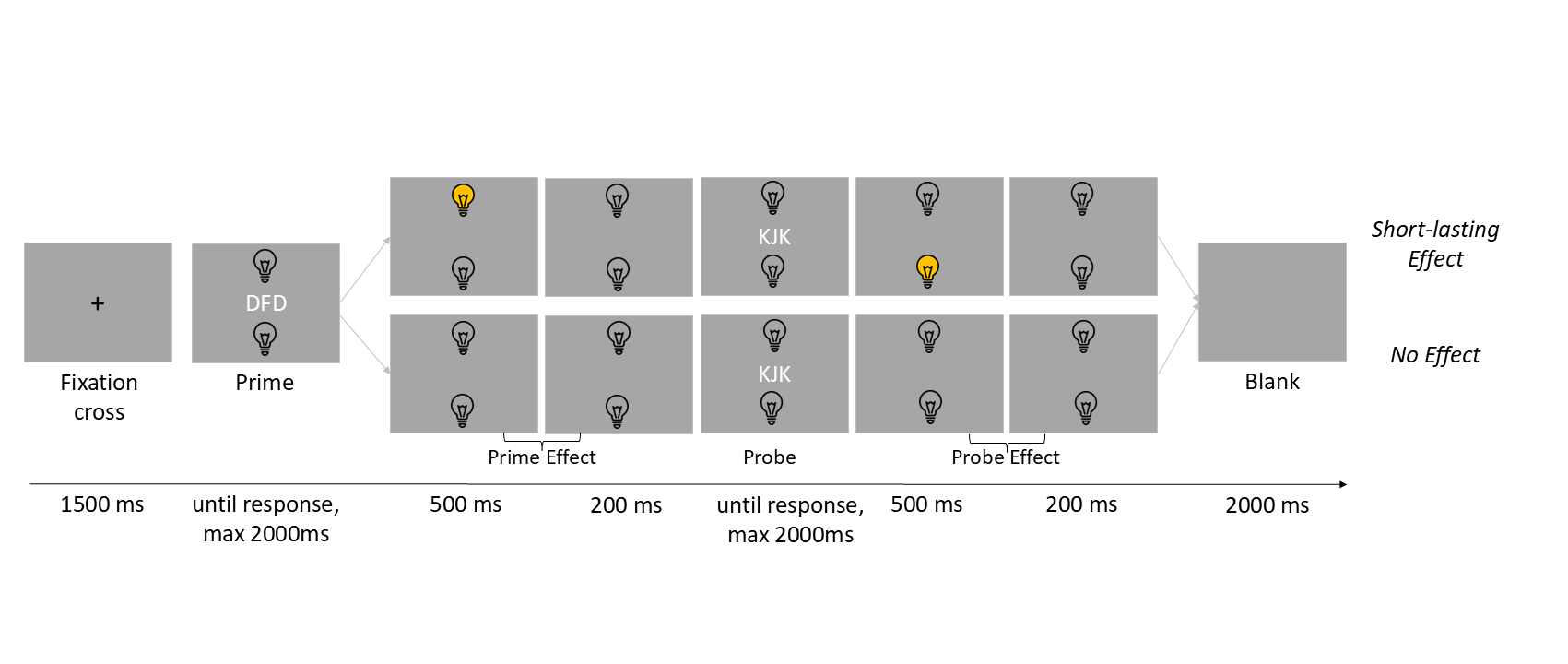
***Note*. Shown are trials with no action effect and short-lasting action effect. Stimuli are not drawn to scale.

**Supplementary 4B**

*Exp4: ANOVA output (RT)*

| Predictor | *df_Num_* | *df_Den_* | *F* | *p* | η^2^_p_ |
| --- | --- | --- | --- | --- | --- |
| (Intercept) | 1 | 61 | 2763.97 | .000 | .98 |
| Response Relation | 1 | 61 | 68.85 | .000 | .53 |
| Distractor Relation | 1 | 61 | 10.29 | .002 | .14 |
| Action Effect Condition | 1 | 61 | 1.60 | .211 | .03 |
| Response Relation x Distractor Relation | 1 | 61 | 16.22 | .000 | .21 |
| Response Relation x Action Effect Condition | 1 | 61 | 0.11 | .739 | .00 |
| Distractor Relation x Action Effect Condition | 1 | 61 | 2.15 | .148 | .03 |
| Response Relation x Distractor Relation x Action Effect Condition | 1 | 61 | 0.28 | .596 | .00 |

*Note.* *df_Num_* indicates degrees of freedom numerator. *df_Den_* indicates degrees of freedom denominator. η^2^_p_ indicates partial eta-squared.

*Lineplot showing the reaction times for all conditions in Experiment 4.*


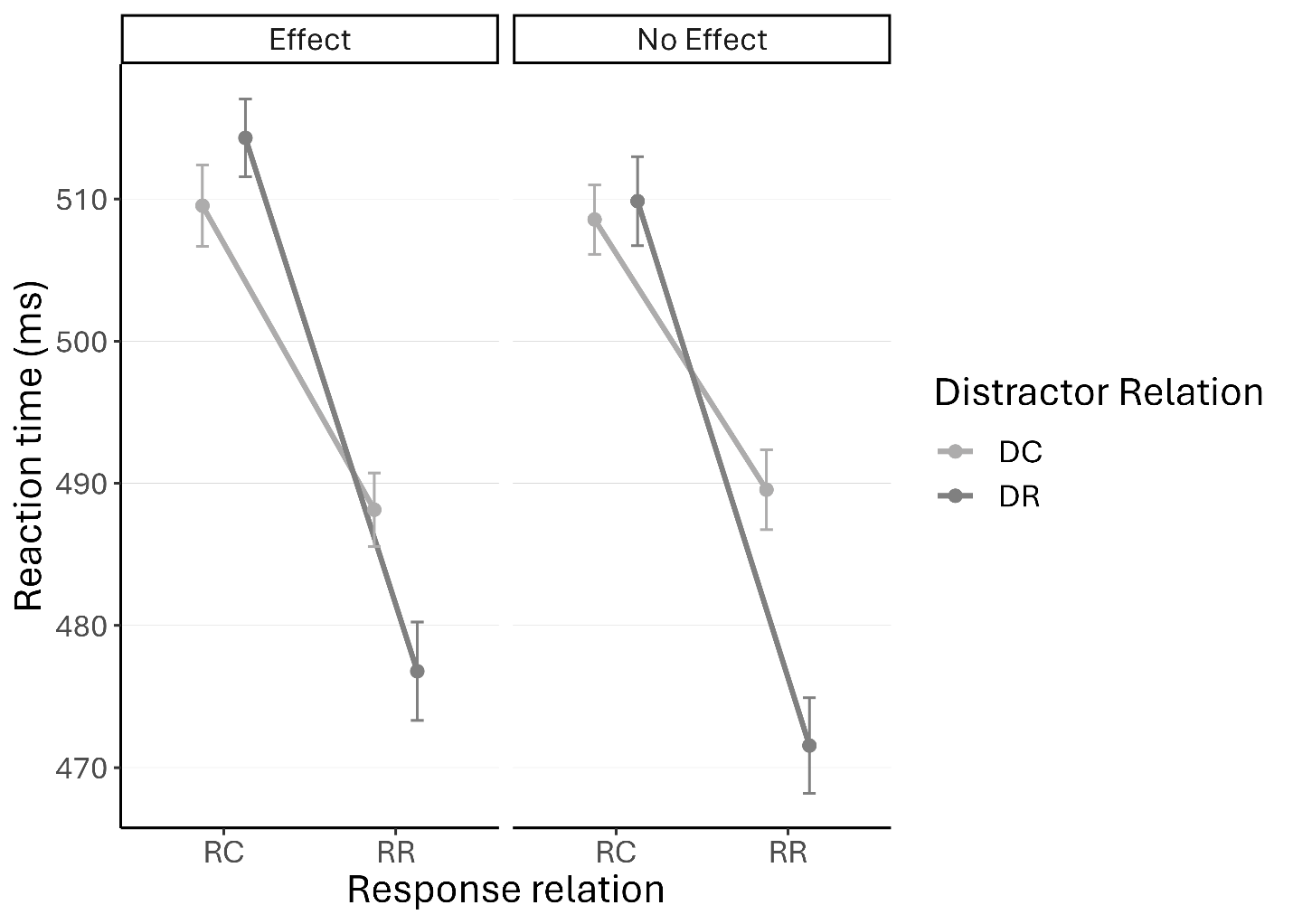


*Note*. Error bars depict the standard error of the mean.

**Supplementary 4C**

*Exp4: ANOVA output (Error rates)*

| Predictor | *df_Num_* | *df_Den_* | *F* | *p* | η^2^_p_ |
| --- | --- | --- | --- | --- | --- |
| (Intercept) | 1 | 61 | 53.08 | .000 | .47 |
| Response Relation | 1 | 61 | 3.64 | .061 | .06 |
| Distractor Relation | 1 | 61 | 3.70 | .059 | .06 |
| Action Effect Condition | 1 | 61 | 0.04 | .840 | .00 |
| Response Relation x Distractor Relation | 1 | 61 | 55.95 | .000 | .48 |
| Response Relation x Action Effect Condition | 1 | 61 | 0.49 | .486 | .01 |
| Distractor Relation x Action Effect Condition | 1 | 61 | 0.41 | .523 | .01 |
| Response Relation x Distractor Relation x Action Effect Condition | 1 | 61 | 0.61 | .439 | .01 |

*Note.* *df_Num_* indicates degrees of freedom numerator. *df_Den_* indicates degrees of freedom denominator. η^2^_p_ indicates partial eta-squared.

*Lineplot showing the error rates for all conditions in Experiment 4.*
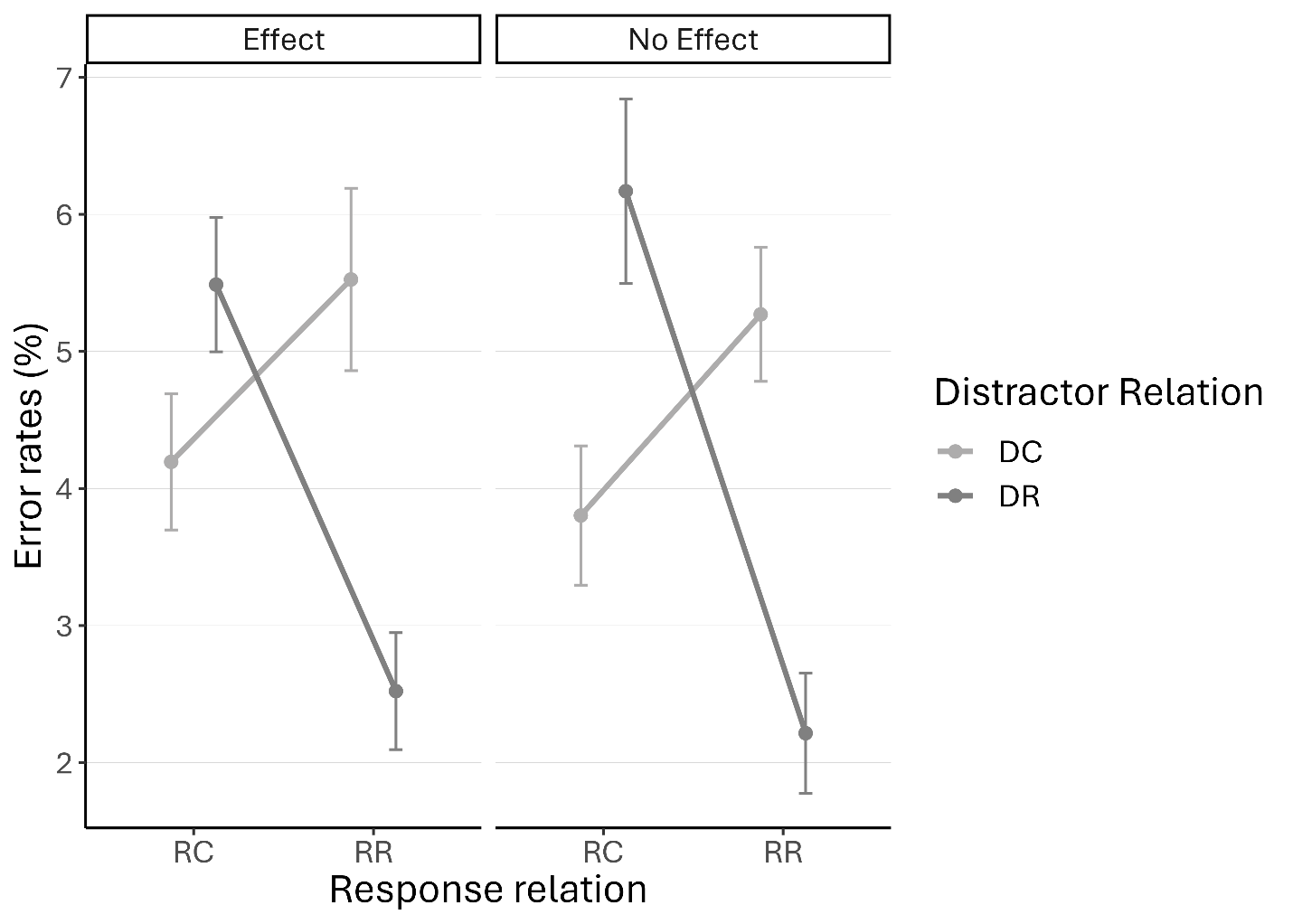


*Note*. Error bars depict the standard error of the mean.

**Supplementary 5**

*Descriptive data for Experiments 1 and 3: Main effect action effect order.*

| Experiment | Action Effect Order | Reaction Times (ms) |
| --- | --- | --- |
| Exp. 1 | ER | 483.94 |
|  | EC | 481.03 |
| Exp. 3 | ER | 461.70 |
|  | EC | 459.94 |

*Note*. Action effect order refers to the relation of action effects in trial n-1 and trial n (ER = Effect Repetition, EC = Effect Change). Experiment 1 looked at long-lasting action effect versus no action effect and Experiment 2 looked at long-lasting action effect versus short-lasting action effect.
